# Spica Prunellae extract inhibits the proliferation of human colon carcinoma cells via the regulation of the cell cycle

**DOI:** 10.3892/ol.2013.1512

**Published:** 2013-08-05

**Authors:** WEI LIN, LIANGPU ZHENG, QUNCHUAN ZHUANG, ALING SHEN, LIYA LIU, YOUQIN CHEN, THOMAS J. SFERRA, JUN PENG

**Affiliations:** 1 Academy of Integrative Medicine, Fujian University of Traditional Chinese Medicine, Fuzhou, Fujian 350122, P.R. China; 2 Fujian Key Laboratory of Integrative Medicine on Geriatrics, Fujian University of Traditional Chinese Medicine, Fuzhou, Fujian 350122, P.R. China; 3 Rainbow Babies and Children's Hospital, Case Western Reserve University School of Medicine, Cleveland, OH 44106, USA

**Keywords:** Spica Prunellae, colorectal cancer, herbal medicine, proliferation, cell cycle

## Abstract

Spica Prunellae has long been used as a significant component in numerous traditional Chinese medicine (TCM) formulas to clinically treat cancers. Previously, Spica Prunellae was shown to promote cancer cell apoptosis and inhibit angiogenesis *in vivo* and *in vitro*. To further elucidate the precise mechanism of its tumoricidal activity, the effect of the ethanol extract of Spica Prunellae (EESP) on the proliferation of human colon carcinoma HT-29 cells was elucidated and the underlying molecular mechanisms were investigated. The proliferation of HT-29 cells was evaluated using 3-(4, 5-dimethyl-thiazol-2-yl)-2,5-diphenyltetrazolium bromide (MTT) and colony formation analyses. The cell cycle was determined using fluorescence-activated cell sorting (FACS) with propidium iodide (PI) staining. The mRNA and protein expression of cyclin-dependent kinase 4 (CDK4) and cyclin D1 was examined using RT-PCR and western blotting, respectively. EESP was observed to inhibit HT-29 viability and survival in a dose- and time-dependent manner. Furthermore, EESP treatment blocked G_1_/S cell cycle progression and reduced the expression of pro-proliferative cyclin D1 and CDK4 at the transcriptional and translational levels. Altogether, these data suggest that the inhibition of cell proliferation via G_1_/S cell cycle arrest may be one of the mechanisms through which Spica Prunellae treats cancer.

## Introduction

Colorectal carcinoma (CRC) is one of the most common cancers with over one million new cases worldwide every year (1,2). Although surgical resection and complete removal of the tumor offers the best prognosis for long-term survival, ~20% of CRC patients present with metastatic disease at the time of the diagnosis, and surgery may not always extirpate the recurrence of advanced CRC (3). Therefore, chemotherapy remains one of the major non-surgical therapeutic approaches for patients with advanced CRC. Despite the steady progress that has been made in the field of chemotherapy and targeted therapy, the majority of patients that undergo chemotherapy experience severe, debilitating and lethal adverse drug events that considerably outweigh the benefits (3–5). In addition, the long-term administration of currently used chemotherapeutic agents usually generates drug resistance (6). These problems highlight the urgent requirement for the development of novel anticancer agents.

Natural products, including traditional Chinese medicine (TCM), have received great interest as they have relatively few side-effects and have long been used clinically as a significant alternative remedy for a variety of cancers (7–14). Spica Prunellae, the fruit-spikes of the perennial plant, *Prunella vulgaris L*., is a medicinal herb that is widely distributed in Northeast Asia. As a well-known Chinese folk medicinal herb with pharmacological properties of heat-clearing and detoxification, Spica Prunellae is traditionally used to treat poor vision, blood stasis, edema, acute conjunctivitis, lymphatic tuberculosis, scrofula, acute mastitis, mammary gland hyperplasia, thyromegaly and hypertension (15). Furthermore, Spica Prunellae has also been employed as a significant component in several TCM formulas for the clinical treatment of several types of cancer, including CRC (16,17). Although we previously reported that the extract of Spica Prunellae promotes the apoptosis of human colon carcinoma cells and displays anti-angiogenic activity *in vitro*(18,19), the mode of its anticancer action remains largely unknown. To further elucidate the mechanism of the tumoricidal activity of Spica Prunellae, the present study evaluated the effect of the ethanol extract of Spica Prunellae (EESP) on the proliferation of human colon carcinoma HT-29 cells and investigated the underlying molecular mechanisms.

## Methods

### Materials and reagents

Dulbecco's modified Eagle's medium (DMEM), fetal bovine serum (FBS), penicillin-streptomycin, trypsin-ethylenediaminetetraacetic acid (EDTA) and TRIzol reagent were purchased from Invitrogen Corporation (Carlsbad, CA, USA). SuperScript II reverse transcriptase was provided by Promega Corporation (Madison, WI, USA). Cyclin D1, cyclin-dependent kinase 4 (CDK4), β-actin antibodies and horseradish peroxidase (HRP)-conjugated secondary antibodies were obtained from Cell Signaling Technology (Danvers, MA, USA). All the other chemicals that were used, unless otherwise stated, were obtained from Sigma-Aldrich Corporation (St. Louis, MO, USA).

### Preparation of EESP

A total of 500 g Spica Prunellae was extracted with 5,000 ml 85% ethanol using a reflux method and filtered. The ethanol solvent was evaporated on a rotary evaporator (RE-2000; Shanghai Yarong Biochemical Instrument Factory, Shanghai, China) and concentrated to a relative density of 1.05. Dried powder EESP was obtained by spray desiccation using a spray dryer (B-290; Büchi Labortechnik AG, Flawil, Switzerland). The stock solution of EESP was prepared by dissolving the EESP powder in 50% dimethyl sulfoxide (DMSO) to a stock concentration of 500 mg/ml, and the working concentrations were made by diluting the stock solution in the cell culture medium. The final concentration of DMSO in the medium for all the cell experiments was <0.5%.

### Cell culture

Human colon carcinoma HT-29 cells were obtained from the Cell Bank of the Chinese Academy of Sciences (Shanghai, China). The cells were grown in DMEM containing 10% (v/v) FBS, 100 U/ml penicillin and 100 μg/ml streptomycin, in a 37°C humidified incubator with 5% CO_2_. The cells were subcultured at 80–90% confluency.

### Cell viability evaluation

Cell viability was assessed using a 3-(4,5-dimethyl-thiazol-2-yl)-2,5-diphenyltetrazolium bromide (MTT) colorimetric assay. The HT-29 cells were seeded into 96-well plates at a density of 1×10^4^ cells/well in 0.1 ml medium. The cells were treated with various concentrations of EESP for different periods of time. At the end of the treatment, 100 μl MTT [0.5 mg/ml in phosphate-buffered saline (PBS)] was added to each well and the samples were incubated for an additional 4 h at 37°C. The purple-blue MTT formazan precipitate was dissolved in 100 μl DMSO. The absorbance was measured at 570 nm using an ELISA reader (ELX800; BioTek, Winooski, VT, USA).

### Colony formation

The HT-29 cells were seeded into 6-well plates at a density of 2×10^5^ cells/well in 2 ml medium. Following the treatment with various concentrations of EESP for 24 h, the cells were harvested and diluted in fresh medium in the absence of EESP and then reseeded into 6-well plates at a density of 1×10^3^ cells/well. Following an eight-day incubation period in a 37°C humidified incubator with 5% CO_2_, the formed colonies were fixed with 10% formaldehyde, stained with 0.01% crystal violet and counted. Cell survival was calculated by normalizing the survival of the control cells as 100%.

### Cell cycle analysis

The cell cycle analysis was performed by flow cytometry using a fluorescence-activated cell sorting (FACS) caliber (Becton Dickinson, San Jose, CA, USA) and propidium iodide (PI) staining. Subsequent to being treated with various concentrations of EESP for 24 h, the HT-29 cells were harvested and adjusted to a concentration of 1×10^6^ cells/ml, then fixed in 70% ethanol at 4°C overnight. The fixed cells were washed twice with cold PBS and then incubated for 30 min with RNase (8 μg/ml) and PI (10 μg/ml). The fluorescent signal was detected through the FL2 channel and the proportion of DNA that was present in the various phases was analyzed using ModfitLT Version 3.0 (Verity Software House, Topsham, ME, USA).

### RNA extraction and RT-PCR analysis

The HT-29 cells were seeded into 6-well plates at a density of 2×10^5^ cells/well in 2 ml medium. The cells were treated with various concentrations of EESP for 24 h. Total RNA was isolated using TRIzol reagent. Oligo(dT)-primed RNA (1 μg) was reverse transcribed with SuperScript II reverse transcriptase according to the manufacturer's instructions. The obtained cDNA was used to determine the amount of CDK4, cyclin D1 and glyceraldehyde 3-phosphate dehydrogenase (GAPDH) mRNA using PCR with Taq DNA polymerase (Fermentas, Waltham, MA, USA). GAPDH was used as an internal control. The primers that were used for amplification of the CDK4, cyclin D1 and GAPDH transcripts were as follows: CDK4 forward, 5′-CAT GTA GAC CAG GAC CTA AGC-3′ and reverse, 5′-AAC TGG CGC ATC AGA TCC TAG-3′; cyclin D1 forward, 5′-TGG ATG CTG GAG GTC TGC GAG GAA-3′ and reverse, 5′-GGC TTC GAT CTG CTC CTG GCA GGC-3′; and GAPDH forward, 5′-GT CAT CCA TGA CAA CTT TGG-3′ and reverse, 5′-GA GCT TGA CAA AGT GGT CGT-3′.

### Western blotting

The HT-29 cells were seeded into 25-cm^2^ flasks at a density of 2×10^5^ cells/well in 5 ml medium. The cells were treated with various concentrations of EESP for 24 h and then lysed with mammalian cell lysis buffer containing protease and phosphatase inhibitor cocktails. The lysates were resolved in 12% sodium dodecyl sulfate-polyacrylamide gel electrophoresis (SDS-PAGE) and electroblotted. The polyvinylidene difluoride (PVDF) membranes were blocked with 5% skimmed milk and probed with primary antibodies against cyclin D1 (monoclonal, mouse), CDK4 (monoclonal, mouse) and β-actin (polyclonal, rabbit) at 1:1,000 dilution overnight at 4°C and then with the appropriate HRP-conjugated secondary antibody followed by enhanced chemiluminescence detection.

### Statistical analysis

All the data are presented as the mean of three determinations. The data were analyzed using the SPSS package for Windows (version 11.5; SPSS, Inc., Chicago, IL, USA). The statistical analysis of the data was performed with an ANOVA. P<0.05 was considered to indicate a statistically significant difference.

## Results

### EESP inhibits HT-29 cell proliferation

**Figure 1:**
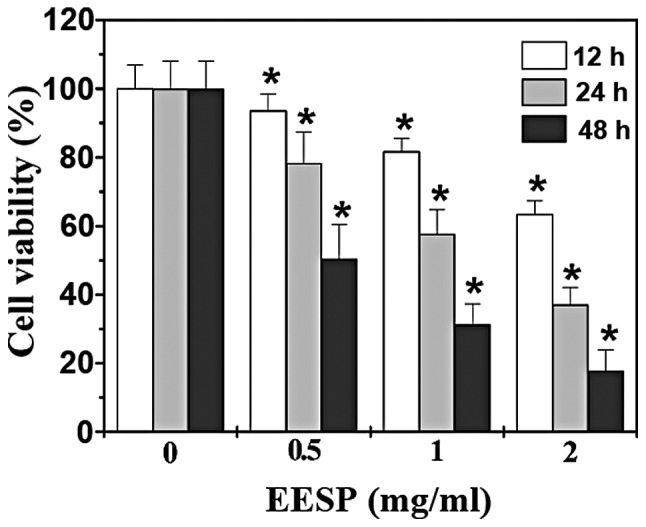
Effect of EESP on the viability of HT-29 cells. The cells were treated with various concentrations of EESP for the indicated time periods. Cell viability was determined using an MTT assay. The data were normalized to the viability of the control cells (100%, treated with 0.5% DMSO vehicle). The data are presented as the mean ± SD (error bars) from three independent experiments. ^*^P<0.05, vs. the control cells. EESP, ethanol extract of Spica Prunellae; MTT, 3-(4,5-dimethyl-thiazol-2-yl)-2,5-diphenyltetrazolium bromide; DMSO, dimethyl sulfoxide.

**Figure 2:**
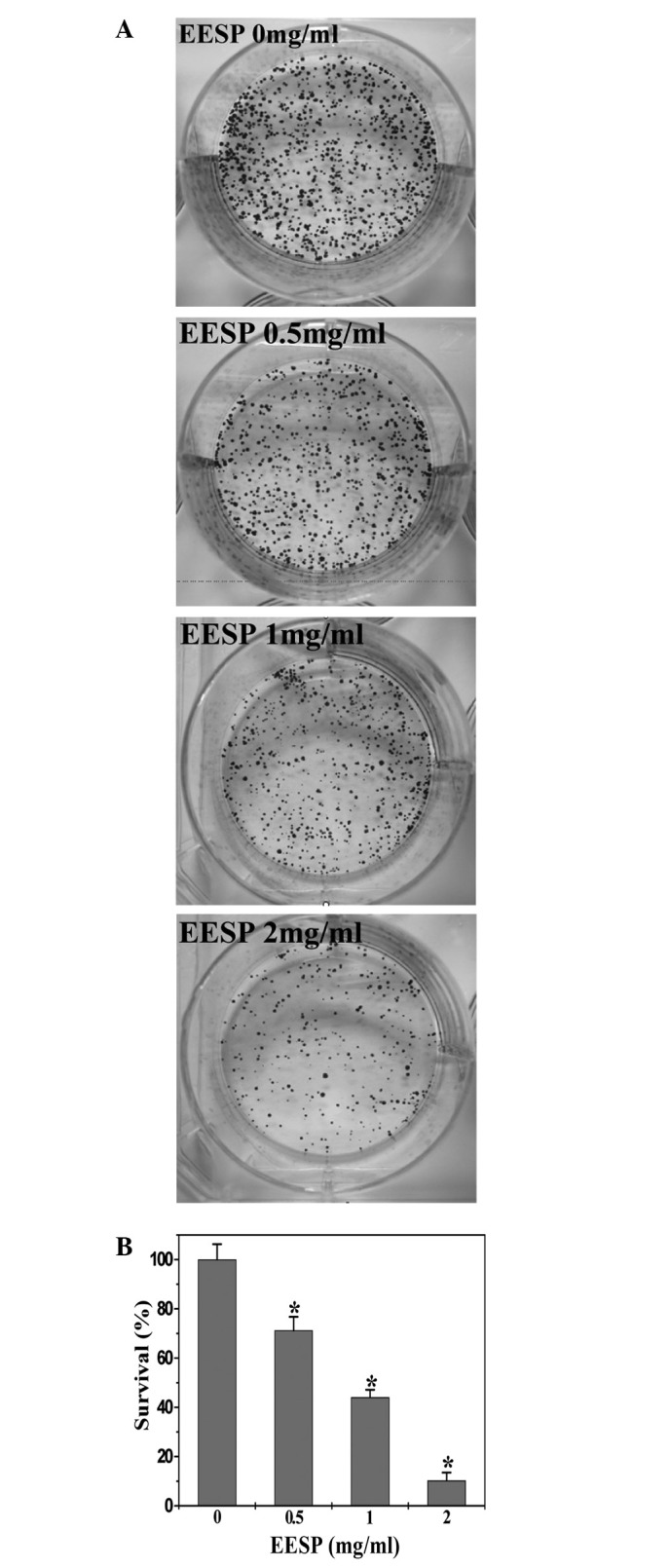
Effect of EESP on cell survival in HT-29 cells. (A) Cell survival was determined using a colony formation analysis. The images are representative of three independent experiments. (B) The data were normalized to the control cells and are shown as the mean ± SD (error bars) from three independent experiments. ^*^P<0.05, vs. the control cells. EESP, ethanol extract of Spica Prunellae.

HT-29 cell viability was examined using an MTT assay to compare the relative number of cells in EESP-treated monolayers with untreated controls. As shown in Fig. 1, treatment with 0.5–2.0 mg/ml EESP for 12, 24 or 48 h, respectively, reduced cell viability by 6.5–49.6, 18.4–68.7 or 36.7–82.2% compared with the untreated control cells (P<0.05). To further verify these results, the effect of EESP on HT-29 cell survival was examined using a colony formation assay. As shown in Fig. 2, EESP treatment dose-dependently reduced the cell survival rate by 28.8–89.8% compared with the untreated control cells (P<0.05). Collectively, these data indicate that EESP inhibited HT-29 cell growth and proliferation in a dose- and time-dependent manner.

### EESP prevents the G_1_/S progression of HT-29 cells

**Figure 3:**
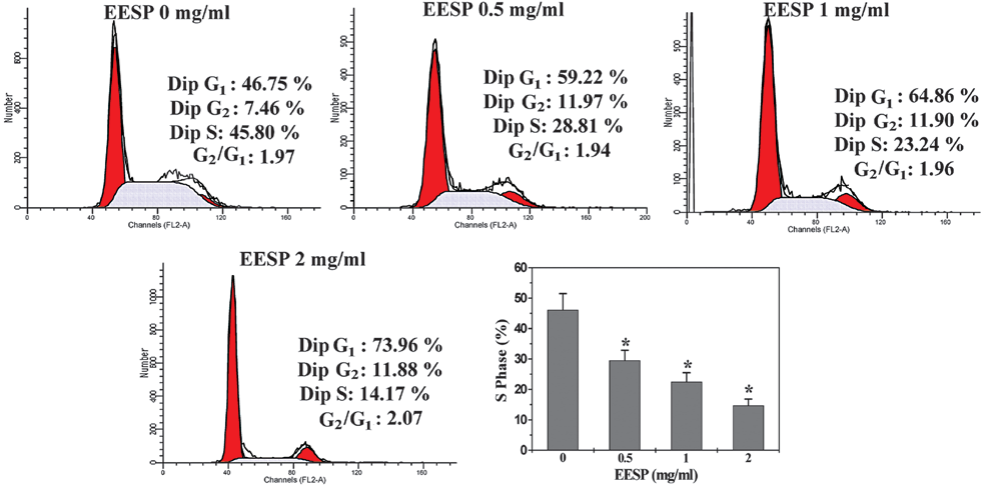
Effect of EESP on cell cycle progression in HT-29 cells. The cells were treated with the indicated concentrations of EESP for 24 h, stained with PI and analyzed using FACS. The proportion of DNA in the S-phase was calculated using ModfitLT Version 3.0 Software. Data are shown as the mean ± SD (error bars) from three independent experiments. ^*^P<0.05, vs. the control cells. EESP, ethanol extract of Spica Prunellae; PI, propidium iodide; FACS, fluorescence-activated cell sorting.

To elucidate the mechanism of the anti-proliferative activity of EESP, its effect on cell cycle progression was examined in HT-29 cells using FACS analysis with PI staining. As shown in Fig. 3, the percentage proportion of S-phase cells following treatment with 0, 0.5, 1 and 2 mg/ml EESP was 46.1±5.3, 29.5±3.3, 22.5±3.0 and 14.7±2.1%, respectively (P<0.05), indicating that the inhibitory effect of EESP on HT-29 cell proliferation was mediated by G_1_/S cell cycle arrest.

### EESP inhibits the expression of cyclin D1 and CDK4 in HT-29 cells

**Figure 4:**
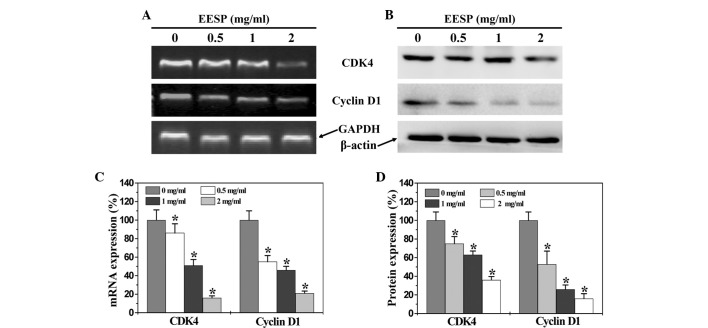
Effect of EESP on CDK4 and cyclin D1 expression in HT-29 cells. The cells were treated with the indicated concentrations of EESP for 24 h. (A) The mRNA levels of CDK4 and cyclin D1 were determined using RT-PCR. (B) The protein expression levels of CDK4 and cyclin D1 were analyzed by western blotting. GAPDH and β-actin were used as the internal controls for the RT-PCR and western blotting assays, respectively. The data are representative of three independent experiments. (C and D) Densitometric analysis. The data were normalized to the mean mRNA or protein expression of the untreated control cells (100%).^*^P<0.05, vs. the control cells. EESP, ethanol extract of Spica Prunellae; GAPDH, glyceraldehyde 3-phosphate dehydrogenase; CDK4, cyclin-dependent kinase 4.

To further explore the mechanism by which EESP inhibited cell proliferation and G_1_/S transition, RT-PCR and western blot analysis were performed to respectively examine the mRNA and protein expression of cyclin D1 and CDK4 in the HT-29 cells. As shown in Fig. 4A and C, EESP treatment significantly and dose-dependently reduced the mRNA expression of pro-proliferative cyclin D1 and CDK4 in the HT-29 cells. The results of the western blot analysis revealed that the protein expression patterns of cyclin D1 and CDK4 were similar to their respective mRNA levels (Fig 4B and D).

## Discussion

Due to drug resistance and the adverse effects of the majority of currently used cancer chemotherapies, natural products receive great interest since they have relatively fewer side-effects and have been used clinically for thousands of years as important alternative remedies for a variety of diseases, including cancer (7–14). One promising medicinal plant is Spica Prunellae, which is widely distributed in Northeast Asia. As a well-known traditional Chinese folk-medicine, it is traditionally used to treat poor vision, blood stasis, edema, acute conjunctivitis, lymphatic tuberculosis, scrofula, acute mastitis, mammary gland hyperplasia, thyromegaly and hypertention (15). In addition, Spica Prunellae has long been employed for the clinical treatment of several types of cancer (16,17). Although we previously reported that Spica Prunellae promotes cancer cell apoptosis and inhibits tumor angiogenesis (18,19), the precise mechanism of its potential tumoricidal activity remains largely unclear. Therefore, prior to the development of Spica Prunellae as an anticancer agent, the mode of its anti-tumor action should be further elucidated.

Cancer cells are characterized by an uncontrolled increase in cell proliferation (20). The presents study therefore investigated the effect of Spica Prunellae on the proliferation of human colon carcinoma HT-29 cells. By using MTT and colony formation analyses, it was demonstrated that EESP dose- and time-dependently inhibited the proliferation of the HT-29 cells. Eukaryotic cell proliferation is primarily regulated by the cell cycle, which consists of four periods: The S phase (DNA synthesis phase), M phase (mitosis), G_1_ phase and G_2_ phase. G_1_/S transition is one of the two main checkpoints of the cell cycle (21), which is responsible for the initiation and completion of DNA replication. Using FACS analysis with PI staining the present study observed that the percentage proportion of S-phase cells was reduced by EESP treatment in a dose-dependent manner, indicating that the inhibitory effect of EESP on HT-29 cell proliferation was mediated by G_1_/S cell cycle arrest. G_1_/S progression is strongly regulated by cyclin D1, which exerts its function by forming an active complex with its major catalytic partners, including CDK4 (22–24). An unchecked or hyperactivated cyclin D1/CDK4 complex often leads to uncontrolled cell division and malignancy (25). By performing RT-PCR and western blot analyses, the present study identified that EESP treatment suppressed the expression of pro-proliferative cyclin D1 and CDK4 in the HT-29 cells at the transcriptional and translational levels.

In conclusion, the present study demonstrated for the first time that Spica Prunellae inhibits the proliferation of cancer cells through G_1_/S cell cycle arrest, which may be one of the mechanisms through which Spica Prunellae exerts its antitumor activity.

## Abbreviations

CRC: colorectal cancer
TCM: traditional Chinese medicine
EESP: ethanol extract of Spica Prunellae
CDK4: cyclin dependent kinase 4
MTT: 3-(4,5-dimethyl-thiazol-2-yl)-2,5-diphenyltetrazolium bromide
DMSO: dimethyl sulfoxide
